# Development of a model webserver for breed identification using microsatellite DNA marker

**DOI:** 10.1186/1471-2156-14-118

**Published:** 2013-12-09

**Authors:** Mir Asif Iquebal, Sarika, Sandeep Kumar Dhanda, Vasu Arora, Sat Pal Dixit, Gajendra PS Raghava, Anil Rai, Dinesh Kumar

**Affiliations:** 1 Centre for Agricultural Bioinformatics, Indian Agricultural Statistics Research Institute, Library Avenue, PUSA, New Delhi 110012, India; 2 Bioinformatics Centre, CSIR-Institute of Microbial Technology, Sector 39A, Chandigarh 160036, India; 3 National Bureau of Animal Genetic Resources, Karnal, Haryana 132 001, India

**Keywords:** Bayesian network, Breed, Goat, Microsatellite, Prediction, Webserver

## Abstract

**Background:**

Identification of true to breed type animal for conservation purpose is imperative. Breed dilution is one of the major problems in sustainability except cases of commercial crossbreeding under controlled condition. Breed descriptor has been developed to identify breed but such descriptors cover only “pure breed” or true to the breed type animals excluding undefined or admixture population. Moreover, in case of semen, ova, embryo and breed product, the breed cannot be identified due to lack of visible phenotypic descriptors. Advent of molecular markers like microsatellite and SNP have revolutionized breed identification from even small biological tissue or germplasm. Microsatellite DNA marker based breed assignments has been reported in various domestic animals. Such methods have limitations *viz*. non availability of allele data in public domain, thus each time all reference breed has to be genotyped which is neither logical nor economical. Even if such data is available but computational methods needs expertise of data analysis and interpretation.

**Results:**

We found Bayesian Networks as best classifier with highest accuracy of 98.7% using 51850 reference allele data generated by 25 microsatellite loci on 22 goat breed population of India. The F_ST_ values in the study were seen to be low ranging from 0.051 to 0.297 and overall genetic differentiation of 13.8%, suggesting more number of loci needed for higher accuracy. We report here world’s first model webserver for breed identification using microsatellite DNA markers freely accessible at http://cabin.iasri.res.in/gomi/.

**Conclusion:**

Higher number of loci is required due to less differentiable population and large number of breeds taken in this study. This server will reduce the cost with computational ease. This methodology can be a model for various other domestic animal species as a valuable tool for conservation and breed improvement programmes.

## Background

Breed of a given species are known to emerge over years during evolution within a specific ecological niche. Each breed is a unique combination of gene in a given gene pool and over the period of time with selection for survival as well as also for productivity due to human intervention. Except cases of commercial crossbreeding under controlled condition, the breed dilution is one of the major problems in sustainability of the breed. The identification of true to breed type animal for conservation purpose is imperative. If we conserve crossbred or admixtured breed, its long term sustenance is compromised as breed is not well adapted over period of time to its native ecological niche. Cross breeding of native goats with exotic breeds of goats (Alpine, Saanen and Boer) has shown poor reproductive performance and high mortality rate in higher grade crosses thus selective breeding of true to the breed type animals is desirable with maintained diversity level for successful conservation and long term sustainability of breed [1]. Such identification tool is also needed to establish breed product’s origin in today’s global market [2].

Though breed descriptor has been developed in India to identify breed but such descriptors cover only “pure breed” type animals which excludes more than 2/3rd of population which are either undefined or admixture [3-5]. In case of close resemblance of phenotype it becomes subjective to identify the breed. Moreover, when degree of admixture is not so conspicuously visible then it is hard to differentiate between true to breed type and “admixtured breed”. Advent of molecular tools like microsatellite and SNP have revolutionized the breed identification even from small samples of biological tissue or germplasm without having ova and semen. In case of semen, ova or embryo the breed cannot be identified as there are no visible breed descriptors.

Microsatellite DNA marker based breed identification has been reported in various domestic animals like cattle [6,7], sheep [8,9], goat [10,11], pig [12], horse [13], dog [14] poultry and rabbit [15]. Such methods have limitations namely, non-availability of allele data in public domain, thus each time all reference breed has to be genotyped which is neither logical nor economical. Even if such data is available but computational methods needs expertise of data analysis and interpretation.

The present work aims at development of a model web server for breed identification where one need not to do genotyping of all referral breeds each time increasing the cost of molecular level identification. In order to achieve this, we have used 51850 allelic data of microsatellite marker obtained from DNA fingerprinting of 22 goat breeds on 25 loci across India. This methodology demonstrates that it can be used as model for other domestic animal species and breed for identification and conservation for long term sustainability endeavor.

## Implementation

### Genomic DNA isolation and creation of data set

Blood samples were collected from a total of 1037 unrelated animals belonging to twenty two different Indian goat breeds. The breeds selected were from diverse geographical regions and climatic conditions with varying utilities and body sizes. Genomic DNA was isolated from the blood samples by using SDS-Proteinase-K method [16,17].

The quality and quantity of the DNA extracted was assessed by Nanodrop 1000 (Thermo Scientific, USA) before further use. A total of 51850 allelic data generated by 25 microsatellite (details can be seen at http://cabin.iasri.res.in/gomi/algorithm.html) loci based DNA fingerprinting on 22 goat breeds *i.e.* Blackbengal, Ganjam, Gohilwari, Jharkhand black, Attapaddy, Changthangi, Kutchi, Mehsana, Sirohi, Malabari, Jamunapari, Jhakarana, Surti, Gaddi, Marwari, Barbari, Beetal, Kanniadu, Sangamnari, Osmanabadi, Zalawari and Cheghu across India were collected. In India, there are 23 registered breeds though FAO reports 32 which are due to vernacular name, geographical name and synonymous name with language diversity.

### Microsatellite DNA markers selection

We followed ISAG (International Society for Animal Genetics) guidelines in marker selection such as (i) at least one marker from each chromosome, (ii) if selected markers are on same chromosome, then must be on different arm of the chromosome, (iii) if still they are in the same arm then distance must be of 50 cM to ensure independent segregation through recombination and (iv) PIC (Polymorphism Information Content) value must be more than 0.5 to ensure higher information of markers in a given population. The data generated using 25 loci *viz*. ILST008, ILSTS059, ETH225, ILSTS044, ILSTS002, OarFCB304, OarFCB48, OarHH64, OarJMP29, ILSTS005, ILSTS019, OMHC1, ILSTS087, ILSTS30, ILSTS34, ILSTS033, ILSTS049, ILSTS065, ILSTS058, ILSTS029, RM088, ILSTS022, OarAE129, ILSTS082 and RM4 (Table 1) was used as standard breed reference at the back end of server [17].

**Table 1:** List of 25 loci along with the primer pairs

**Locus**	**Forward primer**	**Reverse primer**	**Dye**	**Size range**	**No. of observed allele**
ILST008	gaatcatggattttctgggg	tagcagtgagtgaggttggc	FAM	167–195	12
ILSTS059	gctgaacaatgtgatatgttcagg	gggacaatactgtcttagatgctgc	FAM	105–135	14
ETH225	gatcaccttgccactatttcct	acatgacagccaagctgctact	VIC	146–160	9
ILST044	agtcacccaaaagtaactgg	acatgttgtattccaagtgc	NED	145–177	16
ILSTS002	tctatacacatgtgctgtgc	cttaggggtgtattccaagtgc	VIC	113–135	14
OarFCB304	ccctaggagctttcaataaagaatcgg	cgctgctgtcaactgggtcaggg	FAM	119–169	31
OarFCB48	gagttagtacaaggatgacaagaggcac	gactctagaggatcgcaaagaaccag	VIC	149–181	21
OarHH64	cgttccctcactatggaaagttatatatgc	cactctattgtaagaatttgaatgagagc	PET	120–138	10
OarJMP29	gtatacacgtggacaccgctttgtac	gaagtggcaagattcagaggggaag	NED	120–140	14
ILSTS005	ggaagcaatgaaatctatagcc	tgttctgtgagtttgtaagc	VIC	174–190	9
ILSTS019	aagggacctcatgtagaagc	acttttggaccctgtagtgc	FAM	142–162	11
OMHC1	atctggtgggctacagtccatg	gcaatgctttctaaattctgaggaa	NED	179–209	27
ILSTS087	agcagacatgatgactcagc	ctgcctcttttcttgagagc	NED	142–164	11
ILSTS30	ctgcagttctgcatatgtgg	cttagacaacaggggtttgg	FAM	159–179	12
ILSTS34	aagggtctaagtccactggc	gacctggtttagcagagagc	VIC	153–185	15
ILSTS033	tattagagtggctcagtgcc	atgcagacagttttagaggg	PET	151–187	25
ILSTS049	caattttcttgtctctcccc	gctgaatcttgtcaaacagg	NED	160–184	13
ILSTS065	gctgcaaagagttgaacacc	aactattacaggaggctccc	PET	105–135	16
ILSTSO58	gccttactaccatttccagc	catcctgactttggctgtgg	PET	136–188	27
ILSTSO29	tgttttgatggaacacagcc	tggatttagaccagggttgg	PET	148–191	23
RM088	gatcctcttctgggaaaaagagac	cctgttgaagtgaaccttcagaa	FAM	109–147	19
ILSTS022	agtctgaaggcctgagaacc	cttacagtccttggggttgc	PET	186–202	9
OARE129	aatccagtgtgtgaaagactaatccag	gtagatcaagatatagaatatttttcaacacc	FAM	130–175	23
ILSTS082	ttcgttcctcatagtgctgg	agaggattacaccaatcacc	PET	100–136	19
RM4	cagcaaaatatcagcaaacct	ccacctgggaaggccttta	NED	104–127	12

### Data Generation by allele detection and genotyping

PCR products were mixed in ratio of 1:1.5:2:2 of FAM (blue), VIC (green), NED (yellow) and PET (red) labelled respectively after determining the optimal pooling ratio and dilution ratio for a set of primers. In order to ensure size calibration of alleles 0.5 μL of this mixture was combined with 0.3 μL of Liz 500 as internal lane standard (Applied Biosystems) and 9.20 μL of Hi-Di Formamide per sample. The resulting mixture was denatured by incubation for 5 min at 95°C to run on automated DNA sequencer of Applied Biosystems (ABI 3100 Avant). The electropherograms were drawn through Gene Scan and used to extract DNA fragment sizing details using Gene Mapper software (version 3.0) (Applied Biosystems). Generated data is numeric in terms of base pair which is size of each allele along with genotype (combination of allele at every diploid locus). The protocol has been described at http://cabin.iasri.res.in/gomi/tutorial.html. The obtained allelic data were further analysed using FSTAT software (http://www2.unil.ch/popgen/softwares/fstat.htm) to compute relative locus differentiation of each breed in the entire dataset.

### Bayesian networks as classifiers

Classification is a technique to identify class labels for instances based on a set of features (attributes). Building accurate classifiers from pre-classified data is a very active research topic of machine learning and data mining. In last two decades, many classification algorithms have been proposed including Naïve-Bayes, Neural Network (Multilayer Perceptron), Random Forest and Bayesian Network based classifiers.

Naïve-Bayes, an effective classifier is easy to construct as the structure is given a priori *i.e.,* no structure learning procedure is required. It assumes that features are independent of each other. Although this assumption is not realistic, Naïve-Bayes has surprisingly outperformed many sophisticated classifiers over a large number of datasets, especially where the features are not strongly correlated [18]. Bayesian Network (BN) is a kind of unrestricted classifier. A common feature of Naïve Bayes is that the class node is treated as a special node: the parent of all the features. However, BN treats the class nodes as an ordinary node, it is not necessary a parent of all the feature nodes. The learning methods and the performance of BN for classification are well described by Friedman *et al.* in 1999 [19]. It has powerful probabilistic representation for classification. A Bayesian network *B* which is a graphical model that encodes a probability distribution *P*_*B*_(*A*_1_, *A*_2_, …, *A*_*n*_, *C*) from a given training set. The resulting model can be used so that, given a set of attributes *a*_1_, *a*_2_, …, *a*_*n*_, the classifier based on *B* returns the label/class *c* which maximizes the posterior probability, *i.e.*

$$P_{B}\left(c\left|a_{1},a_{2},\ldots ,a_{n}\right)\right)$$

Let *D* = {**u**_1_, **u**_2_, …, **u**_*n*_} denotes the training data set. Here, each **u**_*i*_ is a tuple of the form $\langle a_{1}^{i},a_{2}^{i},\ldots ,a_{n}^{i},c^{i}\rangle$ which assigns values to the attributes *A*_1_, *A*_2_, …, *A*_*n*_ and to the class variable *C*. The log likelihood function, which measures the quality of learned model, can be written as

$$\begin{array}{ll} \mathrm{LL}\left(B\left|D\right)\right)= & \sum_{i=1}^{N}\log P_{B}\left(c^{i}\left|a_{1}^{i},a_{2}^{i},\ldots ,a_{n}^{i}\right)\right)\\ & +\sum_{i=1}^{N}\log P_{B}\left(a_{1}^{i},a_{2}^{i},\ldots ,a_{n}^{i}\right) \end{array}$$

The first term in above equation measures efficiency of network *B* to estimate the probability of a class given set of attribute values. The second term measures how well network *B* estimates the joint distribution of the attributes. Since the classification is determined based on *P*_*B*_(*C*|*A*_1_, *A*_2_, …, *A*_*n*_), only the first term is related to the score of the network as a classifier *i.e.,* its predictive accuracy. This term is dominated by the second term, when there are many observations. As *n* grows larger, the probability of each particular assignment to *A*_1_, *A*_2_, …, *A*_*n*_ becomes smaller, since the number of possible assignments grows exponentially in *n*. In our study, number of feature (*n*) are the number of alleles (two alleles per locus) *i.e.* 50 and the total number of samples is 1037 which includes 22 breeds (classes). Prediction performance of a Bayesian network has also been compared with Multilayer Perceptron [20] and Random forest algorithm [21].

In this study, WEKA machine learning workbench with extensive collection of machine learning algorithms and data pre-processing methods was used for classification and prediction [22].

### Assessment of the prediction accuracy

The best model was selected using various statistical measures *viz*. sensitivity, specificity, precision or positive predictive value (PPV), negative predictive value (NPV), accuracy, false discovery rate (FDR) and Mathew’s correlation coefficient (MCC). Accuracy estimate was obtained using five-fold cross-validation technique [23]. For five-fold cross validation technique, the total observations were divided into five parts. Training was done with four sets of observations and testing with one set. The same was repeated such that each set got the opportunity to fall under the test set. Accuracy for each was recorded and the averages of all these five accuracies were reported. The measures are defined as follows:

$$\begin{array}{c} \begin{array}{cc} \mathrm{Sensitivity}\;\mathrm{or}\;\mathrm{TP}\;\mathrm{Rate}=\mathrm{TP}/\left(\mathrm{TP}+\mathrm{FN}\right)\; & \mathrm{Specificity}=\mathrm{TN}/\left(\mathrm{FP}+\mathrm{TN}\right)\;\\ \mathrm{PPV}=\mathrm{TP}/\left(\mathrm{TP}+\mathrm{FP}\right) & \mathrm{NPV}=\mathrm{TN}/\left(\mathrm{TN}+\mathrm{FN}\right)\\ \mathrm{Accuracy}=\frac{\left(\mathrm{TP}+\mathrm{TN}\right)}{\left(\mathrm{TP}+\mathrm{FP}+\mathrm{TN}+\mathrm{FN}\right)} & \mathrm{FDR}=\mathrm{FP}/\left(\mathrm{FP}+\mathrm{TP}\right) \end{array}\\ \mathrm{MCC}=\frac{\left(\mathrm{TP}*\mathrm{TN}-\mathrm{FP}*\mathrm{FN}\right)}{\sqrt{\left(\mathrm{TP}+\mathrm{FP}\right)\left(\mathrm{TP}+\mathrm{FN}\right)\left(\mathrm{TN}+\mathrm{FP}\right)\left(\mathrm{TN}+\mathrm{FN}\right)}} \end{array}$$

where *TP* = True Positive, *TN* = True Negative, *FP* = False Positive, *FN* = False Negative.

### Web implementation

The server is developed using CGI-Perl script, Hyper Text Markup Language (HTML) and Java Scripts to make it more user-friendly and launched using open source web server software program, Apache. Other models like Random Forest, Multiple Layer Perceptron were logically excluded in web implementation ensuring objectivity of identification accuracy. The user needs to submit the microsatellite allelic data having numeric values in base pairs at http://cabin.iasri.res.in/gomi/gomi.html. The data can also be uploaded either using .csv or .txt format or direct entry in the submission form. The server has tutorial for the users for easy understanding with a sample data at http://cabin.iasri.res.in/gomi/tutorial.html.

## Results and discussion

**Figure 1:**
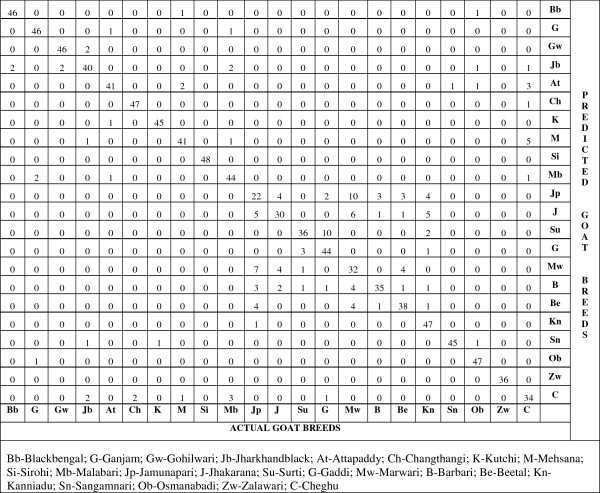
Confusion matrix to show prediction power of BayesNet for each goat breed.

**Figure 2:**
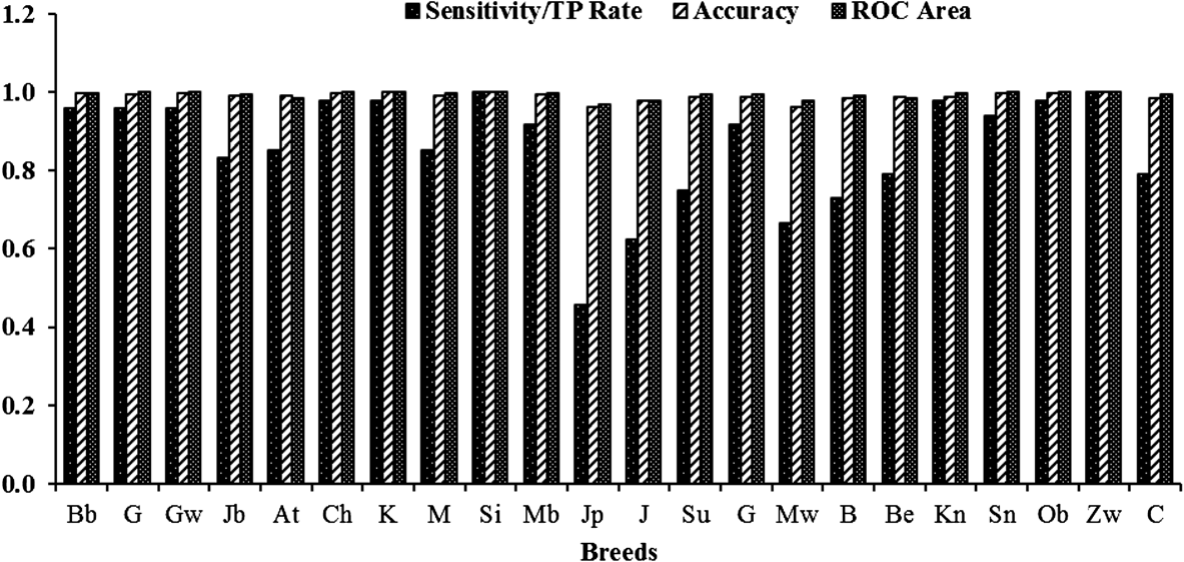
**Graphical representation of various evaluation measures over all the 22 breeds of goat.** Bb-Blackbengal; G-Ganjam; Gw-Gohilwari; Jb-Jharkhandblack; At-Attapaddy; Ch-Changthangi; K-Kutchi; M-Mehsana; Si-Sirohi; Mb-Malabari; Jp-Jamunapari; J-Jhakarana; Su-Surti; G-Gaddi; Mw-Marwari; B-Barbari; Be-Beetal; Kn-Kanniadu; Sn-Sangamnari; Ob-Osmanabadi; Zw-Zalawari; C-Cheghu.

In order to evaluate the performance of Bayesian Network classifier with respect to other popular classifiers such as Naïve Bayes, Multilayer Perceptron and Random Forest, were trained and tested using five-fold cross validation and prediction performance measures were averaged over five test sets. These classifiers were applied over the 51850 allelic/microsatellite data of Indian goat breeds and it has been observed that Bayes Network outperformed other methods (*viz*. Naïve Bayes, Multilayer Perceptron and Random Forest method) with sensitivity (TP Rate), specificity, PPV, NPV, accuracy and MCC values as 0.858, 0.993, 0.860, 0.993, 0.987 and 0.851. The performance of these classifiers is shown in Table 2. Confusion matrix to show prediction power of Bayesian Network for each goat breed is represented in Figure 1. Graphical representation of various evaluation measures (sensitivity or TP Rate, accuracy and ROC area) over all the 22 breeds of goat gives clear picture of the result obtained (Figure 2). The area under ROC (total area equals 1) represents the quality of classification. Higher the value better is the classification which is also evident from our result.

**Table 2:** Performance of different classifiers

**Method**	**Sensitivity**	**Specificity**	**Accuracy**	**MCC**	**FDR**
**Bayes NET**	**0.858**	**0.993**	**0.987**	**0.851**	**0.142**
Naïve Bayes	0.404	0.972	0.946	0.376	0.596
Multilayer-Perceptron	0.450	0.974	0.950	0.424	0.550
Random Forest	0.682	0.985	0.971	0.667	0.318

The best performing classifier is represented in bold.

Similar case of microsatellite data based breed identification using Bayesian method has been found with much higher accuracy for example 99.63% accuracy in five Spanish sheep breed *viz*. Churra, Latxa, Castellana, Rasa-Aragonesa and Merino using 18 microsatellite markers [4]. Similar works have been reported in cattle [24], camel [25] and dog [26].

The novel approach and methodology developed in this study gives higher accuracy which is in similar range of earlier studies in cattle [27]. In some reported cases number of loci needed for breed identification ranged much lower like 3-10 [26,28]. For our study, all the 25 loci were needed which is due to poor differentiation of loci in the breeds. Populations having higher F_ST_ values always needed minimum loci. Contrary to this, population having low F_ST_ needs more number of loci and still the accuracy is compromised. For example, *Murciana* and *Granadina* populations with 25 microsatellites of low F_ST_ value (0.0432) have been reported with just 80% accuracy [29]. Contrary to this, in case of horse, where F_ST_ was having a range of 0.2 to 0.259, the accuracy has been high up to 95%, even with minimum of 3 loci [28].

**Figure 3:**
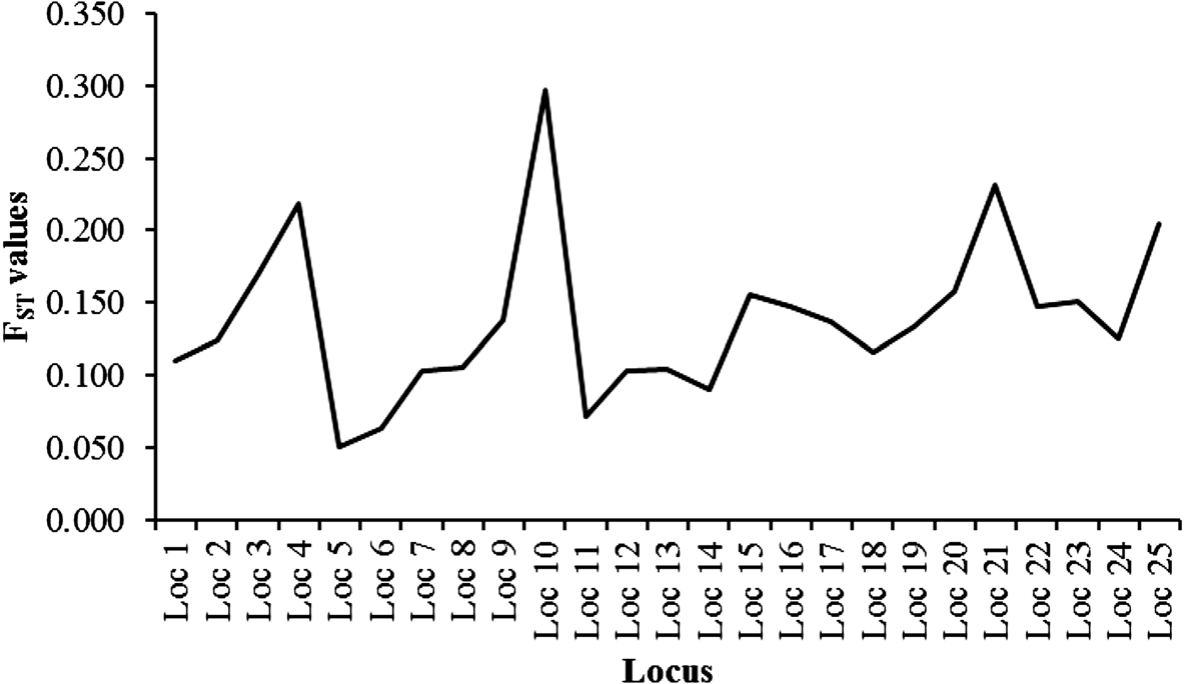
**Graph of F**_**ST **_**values of each locus.**

In case of very low F_ST_ like 0.009, the breed identification accuracy has been reported as low as 39-48% in four breeds. The poor success in correct breed assignment is due to weak genetic differentiation and gene flow between populations [29]. In our study, the F_ST_ values were calculated and were seen to be low ranging from 0.051 at 5th locus to 0.297 at 10th locus and overall genetic differentiation of 13.8%, suggesting more number of loci needed for higher accuracy and we found the expected result in our study (Figure 3). In our observation when loci number was increased this low F_ST_ was compensated for identification accuracy. The relationship between locus differentiation (F_ST_) and accuracy of prediction is proportionate. If F_ST_ value in a given population of locus selected are higher (> 0.10) then number of locus needed is relatively less. If F_ST_ value of loci in a given population is low (<0.05) then more number of loci is required to achieve accuracy [26].

Poor F_ST_ in Indian goat population is already reported in many studies related to goat breeds of India [16,30,31]. This is happening due to unplanned and indiscriminate mating prevalent in breeding region leads to small effective population size or mating between relatives and consequent genetic drift. The general practice of breeding here is to allow few bucks for the whole village/flock [30]. For conservation, proper breeding strategies must be designed by rotating the bucks in their flock since the male:female sex ratio is too low. We found Jhakrana, Marwari and Sirohi having lower sensitivity and MCC (Table 3) which is due to overlapping native breeds of tract where mixing of population prevails in Western India. The low MCC of Jamunapari and Marwari population are obviously expected as lot of allele are getting introduced through immigrant goat breeds in the respective population [30,31].

**Table 3:** Prediction accuracies obtained on twenty two breeds of goat

**Breed**	**Bayes network**				
	**Sensitivity**	**Specificity**	Accuracy (*)	**MCC**	**FDR**
Blackbengal	0.958	0.998	0.996 (0.005)	0.956	0.042
Ganjam	0.958	0.997	0.995 (0.005)	0.946	0.061
Gohilwari	0.958	0.998	0.996 (0.005)	0.956	0.042
Jharkhandblack	0.833	0.994	0.986 (0.006)	0.844	0.130
Attapaddy	0.854	0.997	0.990 (0.006)	0.887	0.068
Changthangi	0.979	0.998	0.997 (0.003)	0.968	0.041
Kutchi	0.978	0.999	0.998 (0.005)	0.977	0.022
Mehsana	0.854	0.996	0.989 (0.006)	0.877	0.089
Sirohi	1.000	1.000	1.000 (0.000)	1.000	0.000
Malabari	0.917	0.993	0.989 (0.006)	0.884	0.137
Jamunapari	0.458	0.980	0.956 (0.003)	0.467	0.476
Jhakarana	0.625	0.990	0.973 (0.011)	0.671	0.250
Surti	0.750	0.995	0.984 (0.008)	0.803	0.122
Gaddi	0.917	0.986	0.983 (0.005)	0.825	0.241
Marwari	0.667	0.976	0.961 (0.021)	0.597	0.429
Barbari	0.729	0.995	0.983 (0.009)	0.790	0.125
Beetal	0.792	0.991	0.982 (0.007)	0.790	0.191
Kanniadu	0.979	0.986	0.986 (0.011)	0.862	0.230
Sangamnari	0.938	0.999	0.996 (0.002)	0.956	0.022
Osmanabadi	0.979	0.996	0.995 (0.006)	0.947	0.078
Zalawari	1.000	1.000	1.000 (0.000)	1.000	0.000
Cheghu	0.791	0.989	0.981 (0.017)	0.763	0.244
**Weighted Avg.**	**0.858**	**0.993**	**0.987**	**0.851**	**0.142**

*The values in parenthesis are the respective standard deviations computer from 5-fold cross validation.

Data in bold represent the weighted average, where weights are the sample sizes for each breed.

## Conclusion

Through the present study, we are reporting first web server for breed prediction with accuracy of more than 98% using 22 goat breeds of India. The number of loci needed is relatively high due to less differentiable population and large number of breeds taken in this study. The web server can be used for other domestic species thus relevant for global use. Further studies are warranted to look for new algorithm to reduce the number of loci in prevailing conditions of large number of breeds and with lower differentiation especially prevailing in “breed melting pot” regions like India and other major diversity regions of the world. This server will reduce the cost with computational ease. This methodology would become a model for all flora and fauna for variety and breed identification required in improvement, conservation, sovereignty issues in trans-border germplasm movement and management.

## Availability and requirements

Webserver can be accessed freely at http://cabin.iasri.res.in/gomi/

**Server Name:**http://cabin.iasri.res.in/

**Project home page:**http://cabin.iasri.res.in/gomi/

**Operating system(s):** e.g. Platform independent

**Programming language:** PERL, Java, PHP

**Other requirements:** Internet connectivity

**License:** No restrictions on non-commercial/Research use

**Any restrictions to use by non-academics:** Non-academicians may contact corresponding author

## Competing interest

The authors declare that they have no competing interests.

## Authors’ contribution

DK, GPSR and AR conceived this study. SPD participated in sample collection and data generation. MAI, S, SKD & VA created the work-flow, web application and performed data analyses. MAI, S and DK drafted the manuscript. All authors read and approved the manuscript.

## References

[B1] Rai B Singh MK Singh SK Goats for meat, milk and fibre: a review Indian J Anim Sci 2005 14 349 355

[B2] Negrini R Nicoloso L Crepaldi P Milanesi E Marino R Perini D Pariset L Dunner S Leveziel H Williams JL Ajmone-Marsan P Traceability of four European Protected Geographic Indication (PGI) breed products using Single Nucleotide Polymorphisms (SNP) and Bayesian statistics Meat Sci 2008 14 1212 1217 10.1016/j.meatsci.2008.05.021 22063859

[B3] Sreenivas D Breeding policy strategies for genetic improvement of cattle and buffaloes in India Vet World 2013 14 455 460 10.5455/vetworld.2013.455-460

[B4] Sharma R Maitra A Singh PK Tatia MS Genetic diversity and relationship of cattle populations of East India: distinguishing lesser known cattle populations and established breeds based on STR markers Springer Plus 2013 14 359 10.1186/2193-1801-2-359 23961421 PMC3733078

[B5] ftp://ftp.fao.org/docrep/fao/010/a1250e/annexes/CountryReports/India.pdf

[B6] Blott SC Williams JL Haley CS Discriminating among cattle breeds using genetic markers Heredity 1999 14 613 619 10.1046/j.1365-2540.1999.00521.x 10383682

[B7] Maudet C Luikart G Taberlet P Genetic diversity and assignment tests among seven French cattle breeds based on microsatellite DNA analysis J Anim Sci 2002 14 942 950 12002331 10.2527/2002.804942x

[B8] Arranz J Bayon Y Primitivo FS Differentiation among Spanish sheep breeds using microsatellites Genet Sel Evol 2001 14 529 542 10.1186/1297-9686-33-5-529 11712973 PMC2705403

[B9] Niu LL Li HB Ma YH Du LX Genetic variability and individual assignment of Chinese indigenous sheep populations (*Ovis aries*) using microsatellites Anim Genet 2011 14 108 111 22221033 10.1111/j.1365-2052.2011.02212.x

[B10] Serrano M Calvo JH Martinez M Marcos-Carcavilla A Cuevas J Gonzalez C Jurado JJ de Tejada PD Microsatellite based genetic diversity and population structure of the endangered Spanish Guadarrama goat breed BMC Genet 2009 14 61 10.1186/1471-2156-10-61 19785776 PMC2761942

[B11] Hoda A Hyka GA Dunner S Obexer-Ruff G Genetic diversity of Albanian goat breeds based on microsatellite markers Arch Zootec 2011 14 607 615 10.4321/S0004-05922011000300049

[B12] Fan B Chen YZ Moran C Zhao SH Liu B Yu M Zhu MJ Xiong TA Li K Individual-breed assignment analyses in swine populations by using microsatellite marker Asian-Aust J Anim Sci 2005 14 1529 1534

[B13] Bjornstad G Roed KH Breed demarcation and potential for breed allocation of horse assessed by microsatellite markers Anim Genet 2001 14 59 65 10.1046/j.1365-2052.2001.00705.x 11421939

[B14] Toskinen MT Bredbadka P A convenient and efficient microsatellite-based assay for resolving parentage in dogs Anim Genet 1999 14 148 149 10.1046/j.1365-2052.1999.00438.x 10376306

[B15] Gotz K Thaller G Assignment of individuals to populations using microsatellites J Anim Breed Genet 1998 14 53 61 10.1111/j.1439-0388.1998.tb00327.x

[B16] Dixit SP Verma NK Aggarwal RAK Vyas MK Rana J Sharma A Genetic diversity and relationship among Indian goat breeds based on microsatellite markers Small Ruminant Res 2012 14 38 45 10.1016/j.smallrumres.2011.11.026

[B17] Kumar S Dixit SP Verma NK Singh DK Pande A Kumar S Chander R Singh LB Genetic diversity analysis of the Gohilwari breed of Indian goat (*Capra hircus*) using microsatellite markers Am J Anim Vet Sci 2009 14 49 57

[B18] Langley P Iba W Thompson K An analysis of Bayesian classifiers In Proceedings of 1992 14 223 228

[B19] Friedman N Geiger D Goldszmidt M Bayesian network classifiers Mach Learn 1997 14 131 161 10.1023/A:1007465528199

[B20] Hassoun MH Fundamentals of artificial neural networks 1995 Cambridge, MA: MIT Press

[B21] Verikas A Gelzinis A Bacauskiene M Mining data with random forests: a survey and results of new tests Pattern Recognit 2011 14 330 349 10.1016/j.patcog.2010.08.011

[B22] Hall M Frank E Holmes G Pfahringer B Reutemann P Witten IH The WEKA data mining software: an update SIGKDD Explorations 2009 14 10 18 10.1145/1656274.1656278

[B23] Efron B Estimating the error rate of a prediction rule: improvement on cross-validation J Am Stat Assoc 1983 14 316 331 10.1080/01621459.1983.10477973

[B24] Canon J Alexandrino P Bessa I Carleos C Carretero Y Dunner S Ferran N Garcia D Jordana J Laloe D Pereira A Sanchez A Moazami-Goudarzi K Genetic diversity measures of local European beef cattle breeds for conservation purposes Genet Sel Evol 2001 14 311 332 10.1186/1297-9686-33-3-311 11403750 PMC2705410

[B25] Mburu DN Ochieng JW Kuria SG Jianlin H Kaufmann B Rege JE Hanotte O Genetic diversity and relationships of indigenous Kenyan camel (*Camelus dromedarius*) populations: implications for their classification Anim Genet 2003 14 26 32 10.1046/j.1365-2052.2003.00937.x 12580783

[B26] Koskinen MT Individual assignment using microsatellite DNA reveals unambiguous breed identification in the domestic dog Anim Genet 2003 14 297 301 10.1046/j.1365-2052.2003.01005.x 12873219

[B27] MacHugh D Loftus RT Cunningham P Bradley DG Genetic structure of seven European cattle breeds assessed using 20 microsatellite markers Anim Genet 1998 14 333 340 10.1046/j.1365-2052.1998.295330.x 9800321

[B28] Bjornstad G Roed KH Evaluation of factors affecting individual assignment precision using microsatellite data from horse breeds and simulated breed crosses Anim Genet 2002 14 264 270 10.1046/j.1365-2052.2002.00868.x 12139505

[B29] Martinez AM Vega-Pla JL Leon JM Camacho ME Delgado JV Ribeiro MN Is the Murciano-Granadina a single goat breed? A molecular genetics approach Arq Bras Med Vet Zootec 2010 14 1191 1198 10.1590/S0102-09352010000500023

[B30] Gour DS Malik G Ahlawat SPS Pandey AK Sharma R Gupta N Gupta SC Bisen PS Kumar D Analysis of genetic structure of Jamunapari goats by microsatellite markers Small Ruminant Res 2006 14 140 149 10.1016/j.smallrumres.2005.07.053

[B31] Kumar D Dixit SP Sharma R Pandey AK Sirohi G Patel AK Aggarwal NK Gour DS Ahlawat SPS Population structure, genetic variation and management of Marwari goats Small Ruminant Res 2005 14 41 48 10.1016/j.smallrumres.2004.11.013

